# Blue Carbon stock in *Zostera noltei* meadows at Ria de Aveiro coastal lagoon (Portugal) over a decade

**DOI:** 10.1038/s41598-019-50425-4

**Published:** 2019-10-07

**Authors:** Ana I. Sousa, José Figueiredo da Silva, Ana Azevedo, Ana I. Lillebø

**Affiliations:** 1 Department of Biology & CESAM – Centre for Environmental and Marine Studies, University of Aveiro, Campus Universitário de Santiago, 3810-193 Aveiro, Portugal; 2 Department of Environment and Planning, University of Aveiro, Campus Universitário de Santiago, 3810-193 Aveiro, Portugal; 3 Department of Physics & CESAM – Centre for Environmental and Marine Studies, University of Aveiro, 3810-193 Aveiro, Portugal

**Keywords:** Ecosystem ecology, Ecosystem services, Wetlands ecology

## Abstract

This work assessed the Blue Carbon (C) stock in the seagrass meadows (*Zostera noltei*) of Ria de Aveiro coastal lagoon (Portugal), and evaluated its spatio-temporal trend over the 2003–2005 to 2013–2014 period. *Zostera noltei* spatial distribution, restricted to intertidal areas in 2014, was mapped by remote sensing using an unmanned aerial vehicle (UAV) and aerial photography. *Zostera noltei* biomass was also monitored *in situ* over a year and its Blue C stock was estimated. By 2014, intertidal meadows covered an area of 226 ± 4 ha and their Blue C stock ranged from 227 ± 6 to 453 ± 13 Mg C. Overall, Ria de Aveiro *Z. noltei* intertidal meadows increased in extent over the 2003–2005 to 2013–2014 period, corroborating the recent declining trend reversal observed in Europe and contrary to the global decline trend. This spatio-temporal shift might be related to a natural adjustment of the intertidal meadows to past human intervention in Ria de Aveiro, namely large-scale dredging activities, particularly in the 1996–1998 period, combined with the more accurate assessment performed in 2014 using the UAV. This recovery contributes to the effective increase of the Blue C stock in Ria de Aveiro and, ultimately, to supporting climate regulation and improving ecosystem health. However, major dredging activities are foreseen in the system’s management plan, which can again endanger the recovery trend of *Z. noltei* intertidal meadows in Ria de Aveiro.

## Introduction

In coastal ecosystems, Blue Carbon (C) corresponds mostly to the C stored in the soil, living biomass (aboveground and belowground), and non-living biomass (e.g. litter) of seagrass meadows, salt marshes, and mangroves^1^. Seagrass meadows play a major role as Blue C sinks, given their ability to sequester large quantities of C in biomass and, more importantly, in the rhizosphere^2–5^, therefore being more efficient C sinks than most terrestrial forests^1^. Seagrass meadows can remove large amounts of atmospheric greenhouse gases such as carbon dioxide (CO_2_)^6,7^, one of the major concerns worldwide. Seagrass meadows also increase sediment trapping, vertical accretion, and plant debris accumulation resulting in increased C storage in the sediment^1,8^. While C is stored for years to decades in plant biomass^1^, it can be stored in the sediment for millennia^9,10^. These high rates of C sequestration and accumulation lead to a large C stock in coastal wetlands and confirm their relevance for climate regulation and climate change mitigation^11,12^, highlighting the need of preserving these habitats. Furthermore, seagrasses are important habitats in the scope of the European Union (EU) Habitats Directive, and valuable indicators of the quality of the marine environment, therefore acknowledged as biological quality elements in the scope of the Water Framework Directive (WFD) and Marine Strategy Framework Directive (MSFD). International conventions such as the Convention for the Protection of the Marine Environment of the North-East Atlantic (“OSPAR Convention”), Helsinki Convention on the Protection of the Marine Environment of the Baltic Sea Area (HELCOM), Barcelona Convention for the Protection of the Mediterranean Sea, and Ramsar Convention on Wetlands, also highlight the need to preserve seagrass meadows. These conventions and regulations are particularly important in a scenario of global seagrass decline, which determines the conservation of these coastal wetlands as critical to assure the C sink capacity of these habitats and their intrinsic ecosystem services (e.g. climate regulation, stabilisation and erosion control, maintenance of nursery populations and habitats).

Despite the environmental and socio-economic importance of seagrass ecological services, these habitats are strongly declining worldwide^13,14^. Natural and anthropogenic drivers have led to coastal wetlands degradation^14^ and sediment-stored C release to the atmosphere and ocean, shifting these ecosystems from C sinks to C sources^14–16^. Some of the main triggers for wetland loss are sediment erosion and physical impacts (e.g. dredging and lugworm burial of seeds and seedlings)^12,17,18^. Herbivores and bioturbators (in high densities) also reduce C storage by increasing microbial mineralization^19^, as well as increasing erosion and re-suspension due to burrowing activities^20–22^. Other anthropogenic pressures include high nutrient loading, sediment contamination, habitat disruption, invasive species, land reclamation, and intense fishing activities^13,23–26^.

Ria de Aveiro, a coastal lagoon in Portugal, is a long-term ecological research (LTER) site (http://www.lter-europe.net/) with several important habitats that justify its inclusion in the Natura 2000 network and classification as a Special Protection Area, comprising areas classified as Sites of Community Importance. Similar to the loss and habitat fragmentation processes observed for seagrass meadows worldwide, those in Ria de Aveiro have declined since the 1980’s to mid 2000’s, mostly through the loss of biodiversity (i.e. loss of *Zostera marina* Linnaeus, 1753, *Stuckenia pectinata* (L.) Börner, 1912, and *Ruppia cirrhosa* (Petagna) Grande, 1918), the loss of subtidal *Zostera noltei* Hornemann, 1832 meadows and reduction of its intertidal meadows^27,28^. The main causes for seagrass decline in Ria de Aveiro were changes in the lagoon hydrodynamics^29–31^, particularly those resulting from harbour related activities (main driving forces)^29^. These activities resulted in the increase of hydromorphological pressures in the lagoon due to dredging of navigable channels, and in changes of hydrodynamic features, such as increased tidal amplitude and current velocity^30–33^. Fishing activities involving motor boating and bait digging in tidal flats^34^ have also been mentioned as pressures because sediment remobilization negatively affects seagrass stability.

Taking into account the importance of seagrass meadows as Blue C sinks, the current work aimed to: 1) assess the current areal extent and distribution of *Z. noltei* meadows at Ria de Aveiro; 2) compare the areal extent and distribution of *Z. noltei* meadows in 2005 and 2014; and 3) estimate the Blue C stock addressing its trend over a decade. Thus, the following null hypotheses were addressed and tested: 1) over the last decade, the spatial extent of seagrass meadows at Ria de Aveiro did not increased or declined, therefore contradicting the global decline trend; and 2) the Blue C stock at Ria de Aveiro *Zostera noltei* meadows in 2014 was not higher than a decade before. To test the outlined hypotheses and address the abovementioned aims, *Z. noltei* areal extent at Ria de Aveiro was assessed and mapped using aerial photography (and an unmanned aerial vehicle) and ground-truth validation; *Z. noltei* biomass and C pools were monitored and quantified monthly, and the Blue C stock was estimated for seagrass meadows.

## Materials and Methods

### Study site

**Figure 1:**
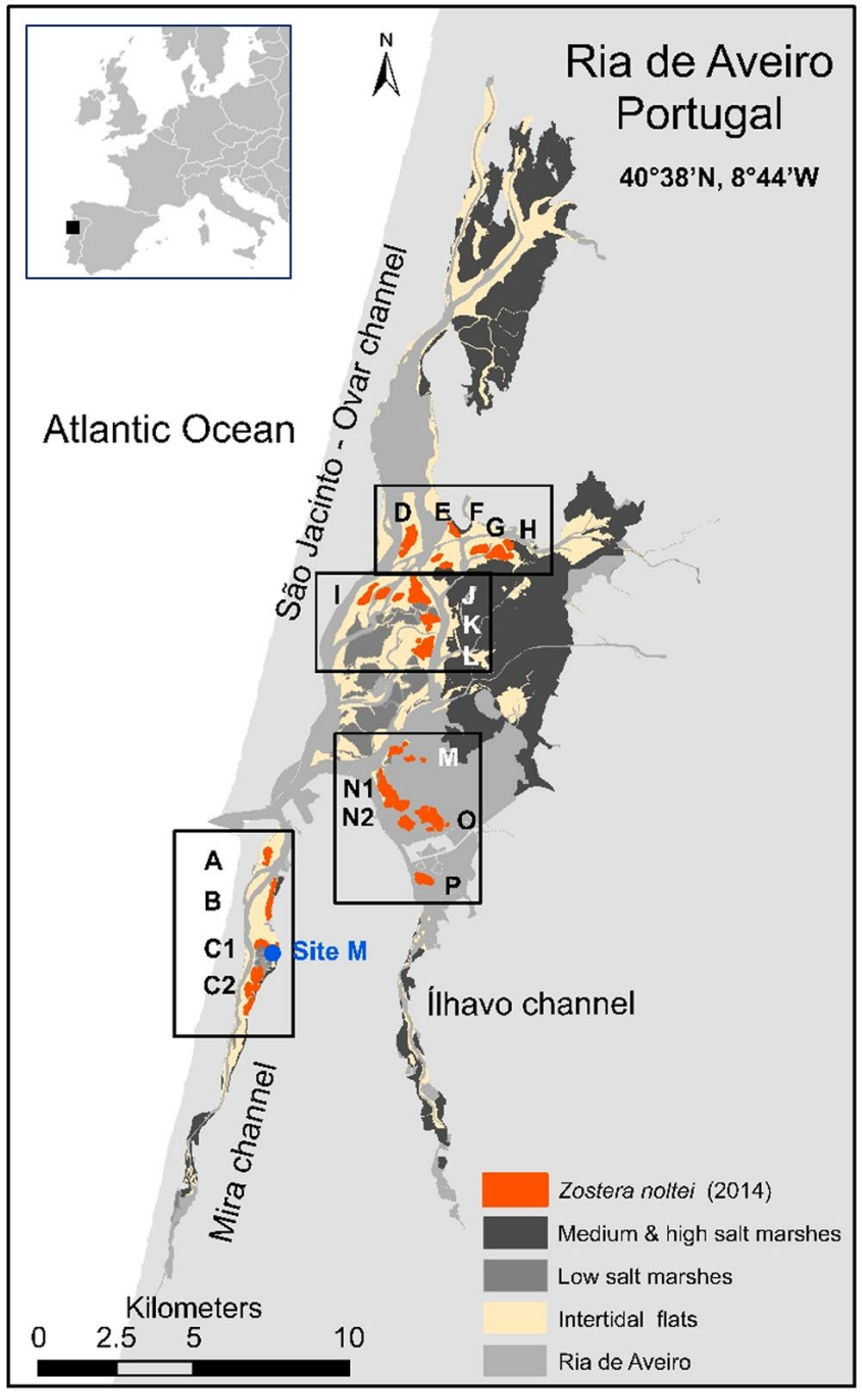
Ria de Aveiro (Portugal) map showing the spatial distribution of salt marshes, seagrass meadows (*Zostera noltei*) and intertidal mudflats. Sampling site for biomass and C stock monitoring at Mira channel (Site M) is indicated as a blue circle. The 18 areas surveyed for *Zostera noltei* spatial distribution and wherein aerial images were acquired are indicated within boxes (A to P). Map generated with ArcGIS 10 (http://www.esri.com/software/arcgis).

Ria de Aveiro is a temperate, shallow, well-mixed coastal lagoon located on the western coast of Portugal (40°38′N, 8°44′W). Its complex geometry includes several branches, inner basins, mudflats, and islands forming four main channels (Mira, Ovar, Espinheiro, and Ílhavo) (Fig. 1). Ria de Aveiro is a mesotidal lagoon, where an engineered inlet channel provides a permanent connection to the Atlantic Ocean, and its semidiurnal tides are the main driver of water circulation within the system.

Before 1990, subtidal and intertidal seagrasses had a massive ecological, economic, and social role in Ria de Aveiro region. Back then, a mixture of seagrasses and macroalgae (“moliço”), was intensively harvested and used as fertilizer in agricultural fields^35^. The commercial harvesting of seagrasses ended about 20 years ago (1998), after a remarkable decrease in the subtidal populations of *Zostera* spp.^32^. Azevedo *et al*.^27^ summarized the available seagrass data for Ria de Aveiro, describing a decrease in coverage in the last decades^31,32^, and mentioning that by 2008 and 2010, in Mira and Ovar channels^28^, respectively, *Zostera noltei* (dwarf eelgrass) was restricted to intertidal areas. Nevertheless, these studies focused on confined seagrass meadows within Ria de Aveiro.

### Mapping of seagrass meadows

To map *Z. noltei* meadows and assess their recent temporal and spatial changes in Ria de Aveiro, aerial photographs were obtained during low tide at selected areas in November 2013 and March 2014 (Fig. 1). These areas were selected for representing the most extensive/larger meadows in 2013–2014 that were also photographed in 2003–2005, and were assessed using an unmanned aerial vehicle (UAV), AscTec Falcon Octocopter, with an RGB camera attached (Sony NEX-5N) equipped with 24-mm lens. For image acquisition, a higher resolution approach was used along the Mira channel (Fig. 1 - areas A, B, C1 and C2). Here, the flights were performed following a predefined path and pattern and the camera was operated at 150–180 m altitude, vertical to the pre-selected points (90° angle). Pixel resolution ranged from 3.8 ± 0.3 to 4.1 ± 0.1 cm. At additional locations (Fig. 1 - areas D to P), lower resolution images were taken at 25–35° of the selected points, from fixed points/locations and capturing panoramic images, at about 200 m altitude. In these areas, pixel resolution ranged from 4.4 ± 0.0 to 5.3 ± 0.1 cm. Based on the pixel resolution, the standard deviation (for each area) and error propagation (for the total area) associated with meadows’ extent estimation were calculated. For both approaches, acquired images were geo-referenced, and combined via a process of image ortho-rectification, mosaicking, and transferring to raster images using the Agisoft PhotoScan^©^ software. The aerial photograph mosaic was then imported to a geographical information system (GIS) using ArcMap (ArcGIS 10), and the boundaries of seagrass meadows were drawn on the map (polygons). The mapped seagrass meadows refer to the external boundaries of the meadows where seagrass relative coverage was up to 100% including some fragmented areas. The areal extent of seagrass meadows (ha) was calculated using ArcGIS. The interpretation of the aerial images recorded in 2013–2014 was validated by ground-truth surveys of the external limits of several of the mapped *Zostera* meadows performed immediately after the UAV flights. These surveys were carried at low tide using a GPS receiver while walking over the external limit of the areas where *Z. noltei* cover was very high (>75%, visual estimation). Data from further field surveys, including local identification of the *Z. noltei* meadows and local photographs, were also included in this validation process.

For the 2003–2005 surveys, aerial images were obtained from a light aircraft, flying at 700–1000 m altitude, several times per year, during low tide. Photographs were taken from the plane using a camera loaded with a 100 ASA colour film and equipped with a 50-mm lens. The images obtained had a tilt angle of up to 35° and their resolutions ranged from 1 m to over 3 m. The visual interpretation of these photographs and the data obtained from ground-truth surveys (performed from 2003 to 2005) were used to validate the seagrass meadows’ boundaries with a 9 m maximum error, by drawing polygons on the map using ArcMap (ArcGIS 10). Following this procedure, the areal extent of seagrass meadows was computed and estimated with an error ranging from 12.2 to 27.5%, depending on the meadow shape (perimeter dimension).

The developed maps for the 2003–2005 and 2013–2014 periods were compared concerning spatial location, extent (area), and temporal or spatial shift. Using ArcGIS, an intersection procedure was performed and the areas that overlapped in time, the new vegetated areas, and the lost meadow areas were identified and mapped. The rates of change (%) for each surveyed area were calculated as described in Waycott *et al*.^14^. Thus, the trajectory of the change, μ (% yr^−1^) was calculated over time (*t*) considering the initial to final surveyed areas (A0 and At, respectively) as μ = [ln(At/Ao)/t]*100. It must be mentioned that technological advances allowed more accurate areal extent estimates in the 2013–2014 assessment than in the 2003–2005 assessment, wherein a larger error was associated with seagrass meadows’ mapping. Nevertheless, the approach used in the present work is relevant in itself, as it addresses the challenge and overcomes the limitations of combining the best available historical data with recent data to study ecological trends. Moreover, this study is the first assessment of seagrasses spatial distribution and extent in the entire Ria de Aveiro lagoon.

### Sampling strategy: *Zostera noltei* biomass monitoring and C pool

*Zostera noltei* biomass was sampled monthly from a well-established meadow at Mira channel (Fig. 1; 40°36′21.57″N, 8°44′15.28″W), for one year (2012–2013) during low tide. At this site, vertical photographs were taken monthly and *Z. noltei* cover was visually assessed, always corresponding to 100% cover. Core-liners were used to sample plant biomass (Ø = 15 cm, area = 177 cm^2^, depth = 12 cm; N = 5) and sediment (Ø = 5 cm, depth = 12 cm; N = 5), and these samples were immediately taken to the laboratory. Here, *Z. noltei* roots and rhizomes were carefully separated from the sediment, rinsed with distilled water, and then dried at 60 °C until constant weight (dry weight, DW). Shoots were subject to the same procedure. *Zostera noltei* epiphytes were not removed as they corresponded to a negligible amount of microalgae. Biomass (g DW m^−2^) was assessed for shoots (aboveground biomass), and for roots and rhizomes (belowground), separately; shoot density (shoots m^−2^) was also quantified. The aboveground and belowground *Z. noltei* biomasses were then ground and homogenised for subsequent analyses.

The top 10 cm sediment layer was air dried, grounded, sieved through a 0.25 mm mesh, and then characterized for total C content. Total C content in the sediment and *Z. noltei* biomasses were quantified (N = 3) in a CHN analyser (Thermo Scientific, Flash2000 Organic Elemental Analyzer, connected to a Thermo Scientific, Delta V Advantage, Isotope Ratio Mass Spectrometer). Correction for inorganic C content in the sediment (C_inorg_) was performed through an acidification protocol, as described in Howard *et al*.^36^. Carbonates (calcium carbonate) were removed through acidification of the sediment (HCl 1 N), which was then gently washed with Milli-Q water, centrifuged, and re-dried at 60 °C. The mass of carbonates in the sediment was then assessed as the mass difference of pre-acidified and acidified sediment, and a correction for the contribution of C (12%) to carbonates was finally performed. The sediment C_inorg_ content was assessed and subtracted from the total C content, to estimate the organic C content (C_org_) in the sediment^36^. The acidification method for the removal of C_inorg_ may remove part of the C_org_, which may lead to the overestimation of C_inorg_ and underestimation of C_org_ in the sediment^37^. For the Blue C stock assessment, only the C_org_ was considered. *Zostera noltei* C_org_ pool or stock (g C m^−2^) was estimated by multiplying biomass (g DW m^−2^) per C content (% DW, or mg g^−1^), while sediment C_org_ stock (g C m^−2^) was calculated by multiplying the C_org_ content (%) per sediment dry bulk density (g DW cm^−3^), considering the volume over a sediment depth of 10 cm. Dry bulk density was assessed at the same site (M) in February and June 2013 (data also included in Sousa *et al*.^38^). The mean dry bulk density was then used for calculations. The term C_org_ stock refers to the amount of C_org_ present at a certain time in a certain area (g C m^−2^) over a particular sediment depth. Total C_org_ stock in *Z. noltei* meadows was calculated integrating both *Z. noltei* aboveground C_org_ stock and sediment C_org_ stock. The C_org_ stock of *Z. noltei* belowground biomass was not included in this calculation because the sediment C_org_ stock already includes the C_org_ of roots and rhizomes. The annual mean C_org_ stock was then used to estimate the total C_org_ stock of seagrass meadows in each mapped area and of all seagrass meadows in the entire Ria de Aveiro system (spatial extent).

To determine the uncertainty of the total C_org_ stock for each assigned seagrass area (*f*), the associated error propagation (*σf*) was computed assuming that *f* depends on “area (value)” (*x*) and “total C_org_ stock” (*y*) and on their associated standard deviations represented by *σ*_*x*_ and *σ*_*y*_, respectively, according to the following equation:

$$\sigma f=\sqrt{{y}^{2}{\sigma }_{x}^{2}+{x}^{2}{\sigma }_{y}^{2}}$$

Total C_org_ stock was extrapolated for all Ria de Aveiro meadows, whose original data (biomass and sediment C_org_) corresponded to well-established meadows (100% cover). Therefore, total C_org_ stock corresponds to the maximum total C_org_ stock if all meadows present 100% cover. Because this is not the most probable scenario in all meadows, either due to new colonised areas (likely to have a lower C_org_ stock) or to habitat fragmentation (reducing the % cover), the C_org_ stock for a potential cover of 50% was estimated. These total C_org_ stock estimates (considering two different % cover) provide a range for the C_org_ stock in the Ria de Aveiro meadows. Although this is a limitation of our estimation method, we assumed that the C_org_ stock was proportional to the seagrass % cover. For the 2005 assessment, the C_org_ stock was estimated considering the C_org_ data obtained for 2013/2014, considering, as this was the best available information, that the C_org_ stock was similar across healthy meadows.

## Results

### Seagrass meadows areal extent at Ria de Aveiro: changes over a decade

**Figure 2:**
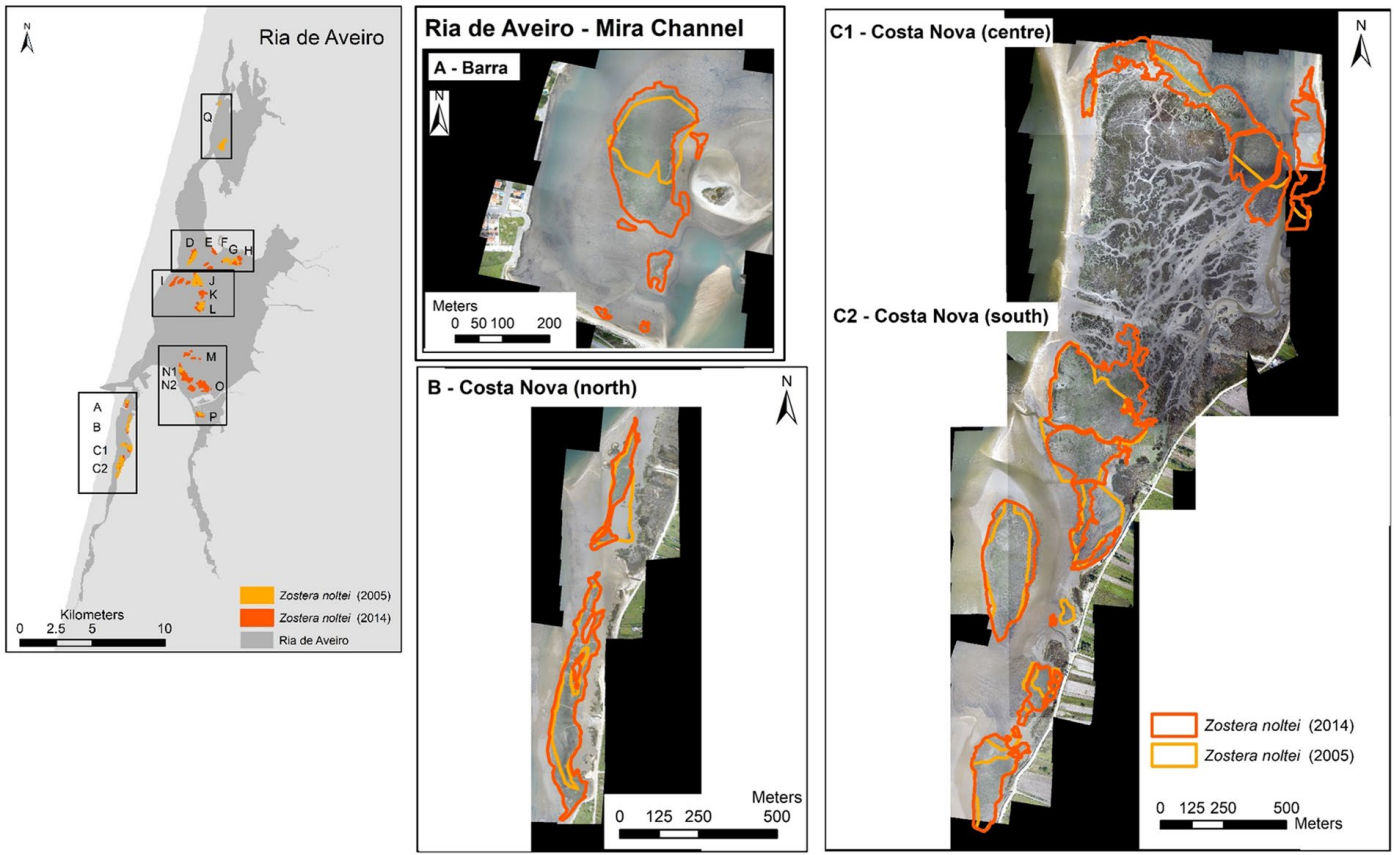
*Zostera noltei* spatial distribution at Ria de Aveiro in 2003–2005 (light orange) and in 2013/2014 (dark orange/red). Letters A to Q correspond to the areas surveyed and mapped in 2003–2005 and/or 2014.Boxes aside the map show the aerial images acquired (UAV) at each area and the seagrass distribution and extent at each survey date. Areas A to C2 were mapped both in 2003–2005 and 2014. Map generated with ArcGIS 10 (http://www.esri.com/software/arcgis). Aerial photograph mosaic performed using Agisoft PhotoScan (https://www.agisoft.com/).

**Figure 3:**
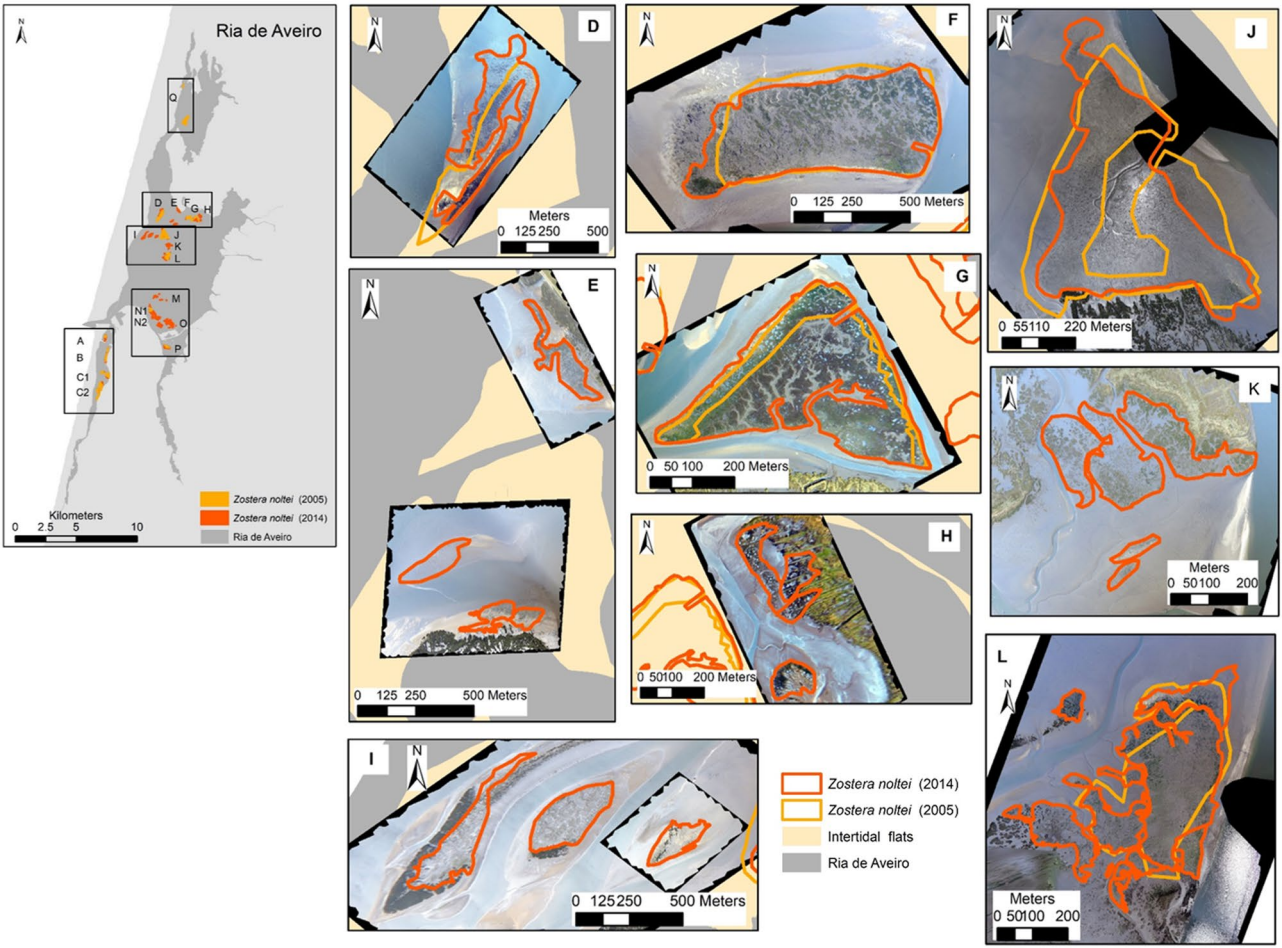
*Zostera noltei* spatial distribution at Ria de Aveiro in 2003–2005 (light orange) and in 2013/2014 (dark orange/red). Boxes aside the map show the aerial images acquired (UAV) at each area and the seagrass distribution and extent at each survey date. Areas D, F, G, J, L correspond to areas surveyed and mapped both in 2003–2005 and 2014; E, H, I, K were mapped only in 2014. Map generated with ArcGIS 10 (http://www.esri.com/software/arcgis). Aerial photograph mosaic performed using Agisoft PhotoScan (https://www.agisoft.com/).

**Figure 4:**
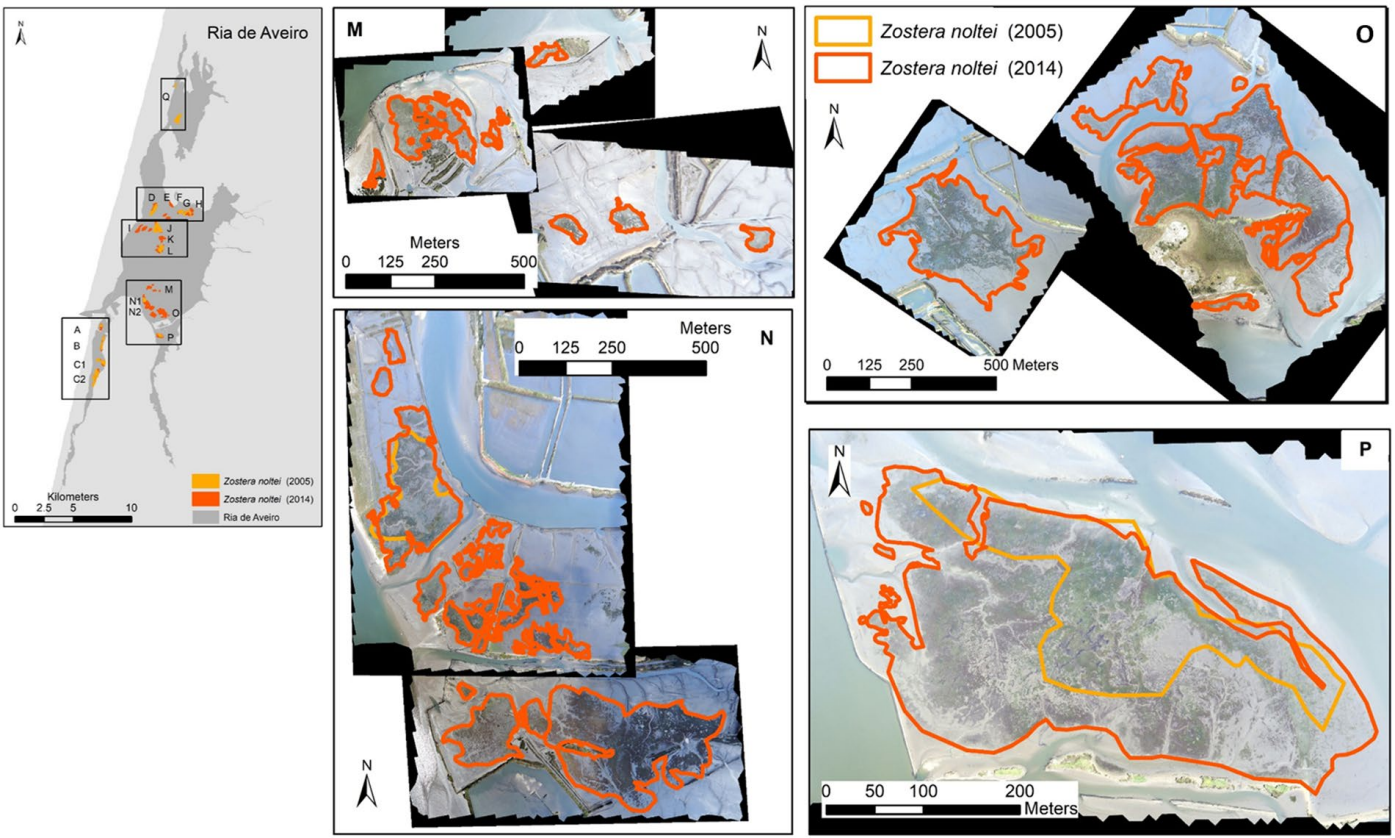
*Zostera noltei* spatial distribution at Ria de Aveiro in 2003–2005 (light orange) and in 2013/2014 (dark orange/red). Boxes aside the map show the aerial images acquired (UAV) at each area and the seagrass distribution and extent at each survey date. Areas N and P correspond to areas surveyed and mapped both in 2003–2005 and 2014; M and O were mapped only in 2014. Map generated with ArcGIS 10 (http://www.esri.com/software/arcgis). Aerial photograph mosaic performed using Agisoft PhotoScan (https://www.agisoft.com/).

Considering the surveyed areas, the *Z. noltei* meadows total area in 2014 was about 226 ± 4 ha (mean ± error propagation) (Figs 2–4). In Mira channel, the areal extent of *Z. noltei* meadows was 40 ± 3 ha (Fig. 2, areas A to C2), in the central north area of Ria de Aveiro it was 120 ± 1 ha (Fig. 3, areas D to L), and in the central south area and Ílhavo channel it was 67 ha (Fig. 4, areas M to P).

**Figure 5:**
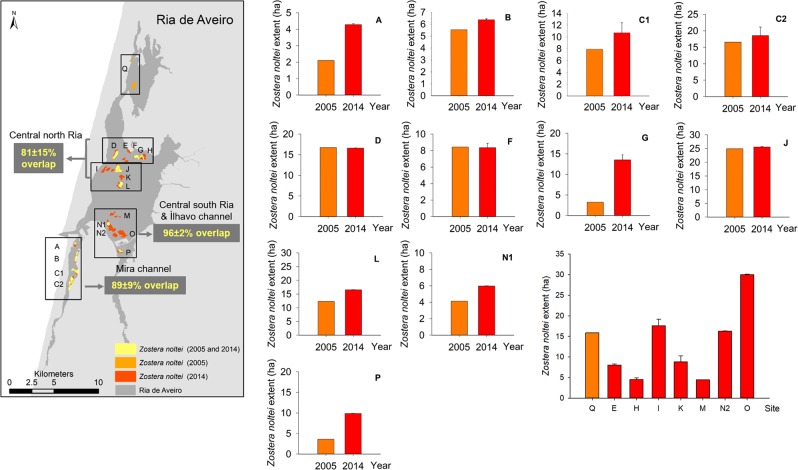
*Zostera noltei* extent (ha) at Ria de Aveiro. Data (mean ± SD) are shown for the surveyed areas in 2005 (light orange) and 2014 (dark orange/red), namely areas A, B, C1, C2, D, F, G, J, L, N1 and P. The meadows overlapped areas (from 2005 and 2014) are shown (yellow) and the mean overlap percentage at each Ria region is indicated in the map. The graph in the lower-right corner shows the *Z. noltei* extent for areas surveyed in 2005 only (area Q) and in 2014 only (areas E, H, I, K, M, N2 and O). Map generated with ArcGIS 10 (http://www.esri.com/software/arcgis). Aerial photograph mosaic performed using Agisoft PhotoScan (https://www.agisoft.com/).

Regarding the areas surveyed in 2013–2014 and in 2003–2005 (A, B, C1, C2, D, F, G, J, L, N1, and P), there was an 1.3-fold increase in the total area of seagrass meadows from 106 to 136 ± 3 ha (mean ± error propagation) (Fig. 5). The surveyed areas showed a general increase in extent, except areas D, F, and J where *Z. noltei* area remained almost stable over time. The surveyed areas in Mira channel increased about 1.4 ± 0.4-fold, from 32 to 40 ha, thus corresponding to a change rate of 2.4% yr^−1^. In the central north area of Ria de Aveiro, there was a 1.1 ± 0.2-fold increase, corresponding to a change rate of 2.4% yr^−1^ (except in area G, where a 4.2-fold increase of the *Z. noltei* area, from 66 to 81 ha, was registered. In the central south area and Ílhavo channel there was a 2.1 ± 0.9-fold increase corresponding to a change rate of 8.0% yr^−1^ (from 8 to 16 ha) (Fig. 5). Overall, the highest increase in *Z. noltei* distribution occurred in areas A, G, and P, all geographically distant. Area Q, located in the north region of Ovar channel and surveyed in 2003–2005 only, had a *Z. noltei* cover area of 16 ha.

The location of *Z. noltei* meadows did not shift over time (2003–2005 vs. 2013–2014) in most of the surveyed areas, and 87 ± 12% of the meadows surveyed in 2003–2005 were still present in 2013–2014. The spatial shift of 13 ± 11% at the surveyed areas corresponded to meadows that were lost or to newly meadows. In Mira channel, about 89 ± 9% (min. 81%, max. 99%) of the meadows recorded in 2003–2005 were also recorded in 2013–2014, being constant over time; 81 ± 15% (min. 62%, max. 99%) of the meadows at the central north region and 96 ± 2% (min. 95%, max. 97%) at the central south and Ílhavo channel were stable (Fig. 5). Globally, the newly colonised and lost meadow areas were small, ranging from 0 to 38% of the surveyed seagrass area. Most of the meadows with a lower increased coverage showed a higher spatial shift over time (B, C2, D, and J but not C1, L, and N1), corresponding to newly colonised and/or lost meadows. Moreover, areas with a higher increase in areal extent showed a reduced spatial shift (A, F, G, and P), corresponding to newly colonised areas. However, it must be taken into account that the mapping methods and accuracy were different between the surveys (2003–2005 and 2013–2014).

### *Zostera noltei* monitoring and characterization

**Figure 6:**
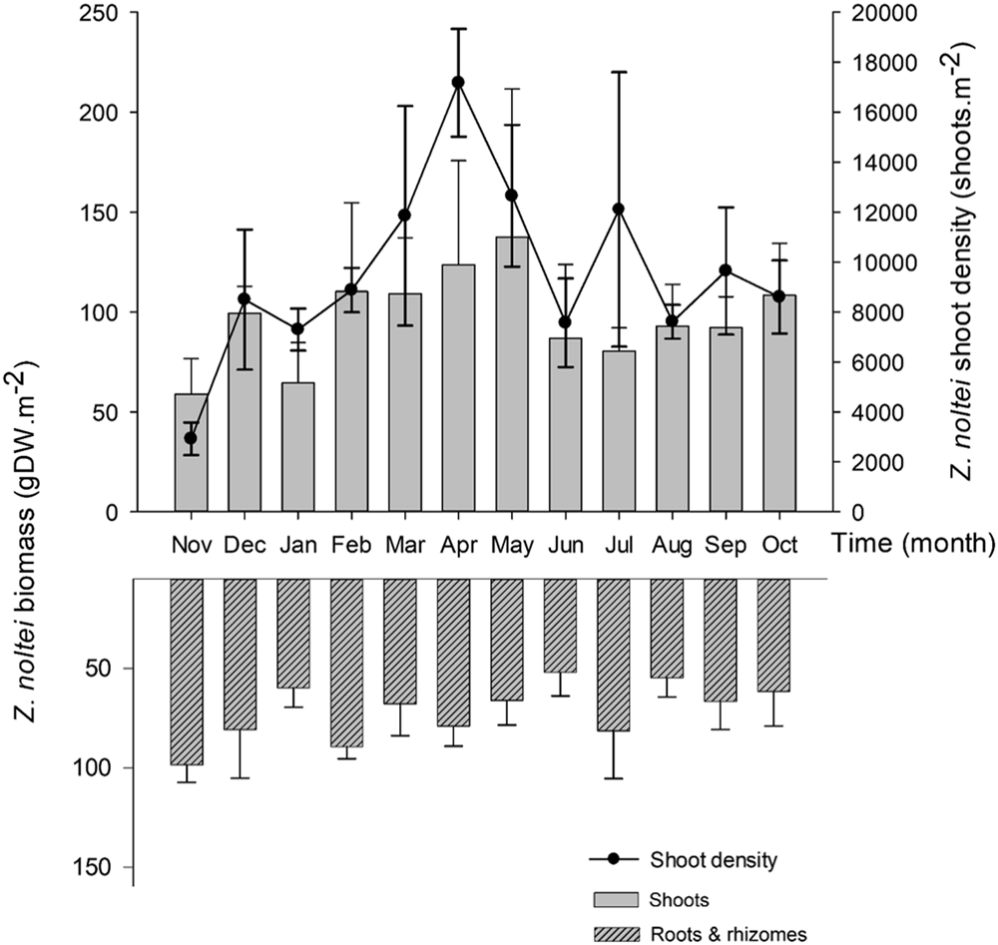
*Zostera noltei* biomass of shoots and roots & rhizomes (A) and shoot density (B), thoughout a year, at site M (Mira channel). Mean values (N = 5) and standard deviation (SD) are shown.

*Zostera noltei* total biomass (including shoots, roots, and rhizomes) reached maximum values in spring (April: 202.8 ± 55.6 g DW m^−2^; May: 203.6 ± 79.9 g DW m^−2^) and minimum values in winter (January: 124.3 ± 26.7 g DW m^−2^) (Fig. 6). Annual mean total biomass was 168.6 ± 25.4 g DW m^−2^. November was the exception, as aboveground seagrass biomass (shoots) was either higher than or similar to belowground biomass. The annual mean shoot density was 9567 ± 4178 shoots m^−2^, ranging from 2926 ± 649 in November to 17170 ± 2160 in April.

*Zostera noltei* C content was slightly higher in the shoots than in the roots and rhizomes, ranging from 37.0 ± 1.0% and 33.5 ± 0.9 in August to 40.2 ± 0.4% and 36.7 ± 0.8% in April, respectively (Fig. S1, supplementary material). Carbon content in the *Z. noltei* sediment showed the lowest value in August (1.7 ± 0.3) and the highest in March (2.1 ± 0.2).

### Blue C pool in *Z. noltei* meadows

The *Z. noltei* C_org_ pool (vegetal material, seagrass) reached the maximum of 75 g C m^−2^ in May (spring) while the minimum value of 46.4 g C m^−2^ was recorded in January (winter). The annual mean *Z. noltei* C_org_ pool was 62.7 ± 10.1 g C m^−2^, with shoots (aboveground biomass) contributing 37.6 ± 8.8 g C m^−2^ and roots and rhizomes (belowground biomass) 25.1 ± 5.4 g C m^−2^ (Table 1). For the sediment, mean dry bulk density was 1.03 ± 0.21 g cm^−3^, total C content ranged from 1.66 ± 0.28 to 2.08 ± 0.23%DW, C_org_ ranged from 1.44 ± 0.08 to 1.84 ± 0.06%DW, and C_inorg_ ranged from 0.19 ± 0.05 to 0.33 ± 0.06%DW (Fig. S1, supplementary material). The annual mean C_org_ pool at the top 10-cm sediment was 162.8 ± 10.9 g C m^−2^ and ranged from 147.3 ± 8.7 g C m^−2^, recorded in November, to 189.0 ± 6.2 g C m^−2^ recorded in March. The total C_org_ pool (including *Z. noltei* biomass and sediment) annual mean was 200.3 ± 15.8 g C m^−2^.

**Table 1:** C_org_ stock of *Zostera noltei* and sediment (0–10 cm depth; mean ± SD, except for total C_org_ stock, wherein mean ± error propagation is shown), over a year (at each sampling date) at site M (Mira channel, Ria de Aveiro, Portugal), recorded in a meadow with 100% coverage.

Time (month)	*Z. noltei* C_org_ stock (g C m^−2^)		Sediment C_org_ stock (g C m^−2^; 10 cm depth)	Total C_org_ stock in *Z. noltei* meadows (g C m^−2^; 10 cm depth)
	Shoots	Roots & rhizomes		
Nov.12	23.5 ± 7.0	35.6 ± 3.2	147.3 ± 8.7	170.8 ± 11.2
Dec.12	39.1 ± 5.3	29.2 ± 8.8	156.7 ± 10.9	195.8 ± 12.1
Jan.13	25.3 ± 7.9	21.1 ± 3.4	169.1 ± 11.3	194.4 ± 13.8
Feb.13	43.8 ± 17.6	31.6 ± 2.1	169.2 ± 5.9	213.0 ± 18.6
Mar.13	40.6 ± 10.4	24.9 ± 5.9	189.0 ± 6.2	229.6 ± 12.1
Apr.13	49.8 ± 20.9	28.1 ± 3.6	168.1 ± 9.9	217.8 ± 23.1
May.13	52.4 ± 28.2	22.6 ± 4.3	157.4 ± 5.5	209.9 ± 28.7
Jun.13	33.2 ± 14.1	17.6 ± 4.0	158.7 ± 9.3	191.9 ± 16.9
Jul.13	31.4 ± 4.5	27.3 ± 8.0	150.8 ± 4.9	182.2 ± 6.7
Aug.13	34.4 ± 7.7	18.4 ± 3.3	163.3 ± 5.3	197.7 ± 9.4
Sep.13	35.7 ± 5.9	23.0 ± 4.9	167.2 ± 9.8	202.9 ± 11.5
Oct.13	41.4 ± 9.9	22.0 ± 6.2	156.7 ± 5.2	198.1 ± 11.2
**C**_**org**_ **stock (mean ± SD)**	**37.6 ± 8.8**	**25.1 ± 5.4**	**162.8 ± 10.9**	**200.3 ± 15.8**

**Figure 7:**
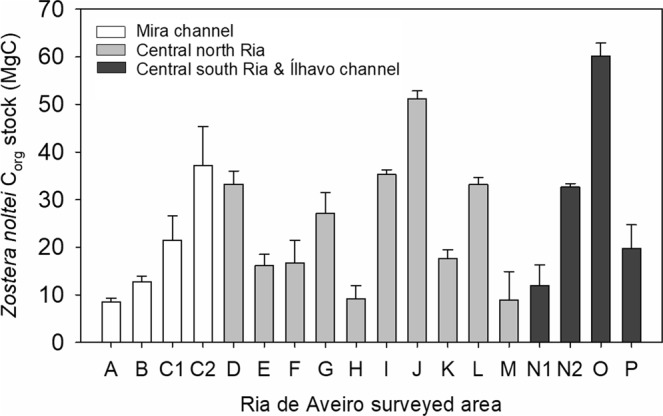
C_org_ stock in *Z. noltei* meadows (seagrass + sediment) at Ria de Aveiro surveyed areas in 2014 (A to L), considering a the maximum (100%) relative cover and 10 cm sediment depth. Mean C_org_ values ± associated error propagation are shown in the graph.

Considering the total C_org_ pool (seagrass and sediment) per unit area and *Z. noltei* spatial extent, a maximum total C_org_ stock of 453.2 ± 12.7 Mg C (mean ± error propagation) was estimated for Ria de Aveiro seagrass meadows in 2014 (considering the hypothetical situation of 100% cover at all meadows), while the maximum total C_org_ stock in 2005 was estimated as 243 Mg C (assuming a 100% cover). Of the total 453 Mg C estimated for 2014, 80 Mg C were stored in the Mira channel meadows, 240 Mg C in the central north area, and 134 Mg C in the central south area and Ílhavo channel (Fig. 7).

## Discussion

The spatial extent of *Z. noltei* in the surveyed areas of Ria de Aveiro was 226 ± 4.1 ha in 2014. About 60% of the seagrass areas were surveyed in both periods (2003–2005 and 2013–2014), and thus we were able to assess and discuss the spatio-temporal evolution of *Z. noltei* meadows at Ria de Aveiro. Over the studied decade, the *Z. noltei* meadows in the surveyed areas recovered, presenting an increase in their spatial extent, from 106 to 136 ha, and there was a global reduced spatial shift in the surveyed meadows (mean 13 ± 11%). Most of the meadows showing a higher increase in their areal extents showed a low spatial shift, indicating that the *Z. noltei* meadows in Ria de Aveiro are stable, which has great ecological relevance, and is reflected in the Blue C stock of these meadows. Nevertheless, when interpreting these spatio-temporal changes one must have in mind that the mapping methods used in the two survey periods (2003–2005 and 2013–2014) were different, meaning that the associated uncertainty was higher in 2003–2005 maps than in 2013–2014 maps. Image resolution was much higher in 2013–2014, increasing the accuracy of the seagrass meadows maps obtained for this period. In addition, polygons defined for seagrass mapping were much more detailed in 2013–2014 than in the previous period due to the high resolution of the 2013–2014 images. The recorded increase in extent and spatial shift trend of *Z. noltei* at Ria de Aveiro over the analysed decade can be due to an effective increase in the meadows extent and/or to an improvement in mapping accuracy through the increase in images’ resolution.

**Figure 8:**
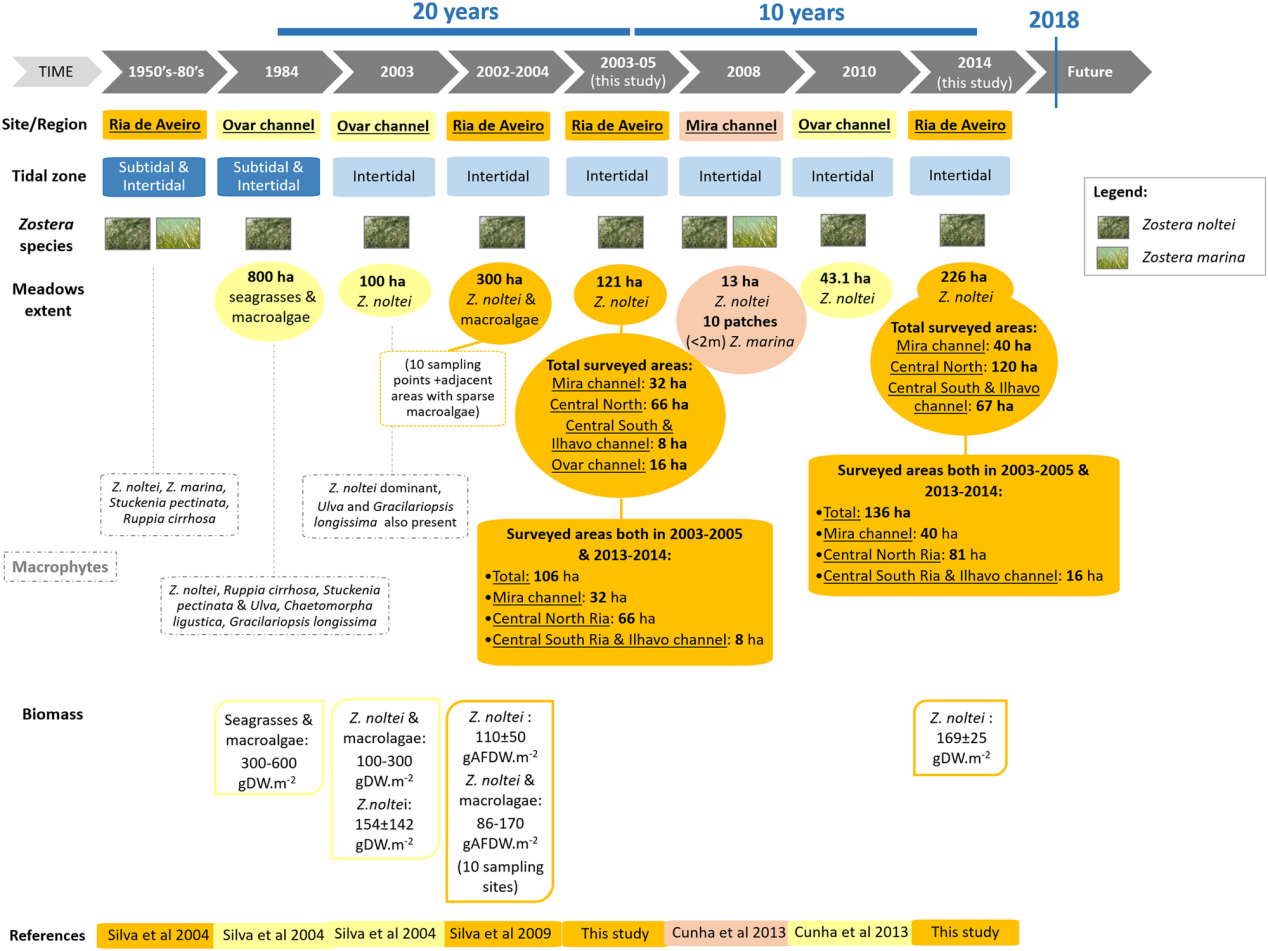
Seagrass meadows spatio-temporal changes at Ria de Aveiro from the 1950’s to 2014. Columns show the data at each surveyed time-range and the corresponding reference. For each site/region, different colours correspond to different surveyed areas of the lagoon (orange: Ria de Aveiro; light yellow: Ovar channel; light orange/pink: Mira channel), and corresponding meadows’ extent, macrophyte’ species and biomass data are indicated. Concerning tidal zone, dark blue refers to subtidal and intertidal zones, while light blue refers to intertidal zone only. References: Silva *et al*.^33^; Silva *et al*.^32^; This study; Cunha *et al*.^29^.

The changes in seagrass meadows at Ria de Aveiro from the 1950’s to 2014 summarized in Fig. 8 were based on our results and on previously published data. Considering the entire Ria de Aveiro system, there was an apparent decrease in seagrass meadows extent from 300 ha in 2002–2004, recorded by Silva *et al*.^31^, to 226 ha in 2014, as obtained in the present study. However, it must be mentioned that Silva *et al*.^31^ data included macroalgae areas adjacent to *Z. noltei*, and not only *Z. noltei* as performed in the 2014 survey. Seagrass meadows located at the Ovar channel were accounted for by Silva *et al*.^31^, but this channel was only partially surveyed in 2014, as area Q was excluded. Therefore, seagrass meadows spatial evolution must be analysed and discussed on comparable areas, as presented at the beginning of this section.

The area of seagrass meadows recorded at the Ovar channel in 2003–2005 (82 ha) was about twice the area recorded by Cunha *et al*.^28^ in 2010 (43 ha). However, the exact locations of the areas surveyed by Cunha *et al*.^28^ were not provided, hindering their comparison. Overall, the total area of seagrass meadows surveyed in 2014 (226 ha) corresponded to the minimum *Z. noltei* extent recorded at Ria de Aveiro. In Mira channel, *Z. noltei* extent in 2008 (13 ha)^28^ was lower than that recorded in the 2003–2005 survey included in the present study, suggesting there might have been episodes of temporal (and spatial) changes and fluctuations of *Z. noltei* meadows over the analysed period (Fig. 8). Overall, there was an increase in the areal extent of the *Z. noltei* meadows mapped in both periods, and newly colonised areas were observed (M, N, and O in the maps displayed in Figs 1 to 3). In addition, most of the meadows mapped in 2005 remained constant or showed an increased areal extents in the 2014 assessment.

Seagrass extent and coverage fluctuations correspond to either fragmentation or recovery of these habitats, which in turn depend on many physical and environmental changes occurring in the system. Increased current speed, loss of fine sediments due to erosion, nutrient loss, and sand burial were pointed out as the main causes for *Z. noltei* decline in Ria de Aveiro in the last decades of the 20^th^ century^31^. Accordingly, sediment resuspension and higher turbidity contributed to the decrease of *Z. noltei* distribution in the Ovar channel from 1984 to 2003^32^. Hydrodynamics, current velocities^39^, sediment stability^25,40,41^, bioturbation^42^, and sedimentation processes^43^ are known to influence seagrass stability.

The most relevant events and changes occurring in Ria de Aveiro coastal lagoon over the last two centuries are summarized in Fig. S2 (supplementary information). These changes are mainly due to human intervention and activities, which led to interrelated effects, and includes modifications of the system hydrodynamics and tidal prism, salinity, and sedimentary and morphological features (please see references in Fig. S2, supplementary information). These pressures that led to the seagrass decline might have been reduced in Ria de Aveiro and the seagrass meadows were able to recover and to compensate the former decline. In fact, the last large dredging operations performed in Ria de Aveiro, involving important areas and affecting the whole system, occurred about 20 years ago (1996–1998) (Fig. S2, supplementary information).

The present study supports that between 2003–2005 and 2013–2014 seagrass meadows showed signs of recovery and resilience after multiple interacting environmental pressures. Although different feedback mechanisms (including seagrass, biotic, and abiotic variables) might be involved in such recovery and resilience signs, it is difficult to address whether a real stable alternative state is achieved by these meadows as the result of feedback mechanisms^44^. The importance of multiple interacting feedback mechanisms on the structure and functioning of seagrass meadows is evident and, according to Maxwell *et al*.^44^, seagrass conservation and recovery management plans might be developed after evaluating and understanding how these meadows are stabilized or disturbed by these feedback mechanisms, therefore increasing seagrass resilience to environmental pressures. It is known that the recovery trajectory of seagrass ecosystems after disturbance depends on the degree of the disturbance and on the resilience of the seagrasses, which in turn depend on the relative timescales of the disturbance, resistance, and recovery processes^45^. These processes are affected by the state and characteristics of the seagrass population, which are tightly dependent on the environmental conditions, feedback mechanisms, scale of degradation, and disturbance history^45^.

There are relevant examples of seagrass meadows recovery in Portuguese (e.g. Mondego estuary^25^ and Ria Formosa^43^) and international systems (e.g. Bay of Santander, Spain^46^, Bourgneuf Bay, France^47^). In addition, a very recent work shows that, in contrast with global estimations, seagrass decline is not generalised in Europe and a recent slowdown and reversal of declining trends have been occurring in some European systems^48^. In the case of Ria de Aveiro, this recovery trend needs special attention because a relevant dredging intervention is foreseen in the system management plan for the near future (2018/2019)^49^. While some meadows are likely to be negatively affected by sediment dredging and deposition, other areas might become favourable for *Z. noltei* establishment, growth, and expansion. Auspicious areas for seagrasses colonisation depend mainly on their exposure to tidal action, elevation, tidal amplitude, tidal prism, and sediment properties. The interaction of these multiple variables, which will also be affected in a mid-long term scale by sea level rise, will therefore determine the areas that are more likely to be colonised by seagrasses.

Concerning *Z. noltei* biomass, the annual mean for the Mira channel (169 ± 25 g DW m^−2^) obtained here is similar to that previously recorded for Ria de Aveiro, namely 154 ± 142 g DW m^−2^ (2003)^32^, 110 ± 50 g AFDW m^−2^ (2002–2004)^31^ and 91 ± 42 DW m^−2^ (shoots^38^) and 46 ± 15 g DW m^−2^ ^38^ (Fig. 6), and it is in accordance with that obtained for Palmones River (Spain)^50^ and Ria Formosa (Portugal)^51,52^. The shoot density in Mira channel (annual mean: 9567 ± 4178 shoots m^−2^) was higher than in other systems, particularly Ria Formosa (southern Portugal), wherein density varied amongst undisturbed and physically disturbed meadows by clam harvesting^51^. Changes in *Z. noltei* shoot density after a disturbance event (physical impact by artificial opening of an inlet) were also observed by Peralta *et al*.^52^. Therefore, different feedback mechanisms may affect the *Z. noltei* morphological response to disturbances.

The *Z. noltei* C content recorded in the present study (annual mean of 39 ± 1% for shoots and 35 ± 1% for roots and rhizomes) was slightly higher, but in the same range as the data recently obtained by Sousa *et al*.^38^. In Ria Formosa, the C content of *Z. noltei* shoots was slightly higher (43–44%)^51^ than in Ria de Aveiro, while in the Palmones River it ranged from 30–40% in the shoots and from 25–40% in the roots and rhizomes.

Blue C stock (C_org_) in the *Z. noltei* meadows of Ria de Aveiro directly depends on the seagrass meadows condition, which is affected by the system stability, in turn influencing the biomass and Blue C stored in both the seagrass biomass and in the sediment. In 2013–2014, *Z. noltei* biomass and C_org_ content in the seagrass and sediment were monitored monthly over a year to examine seasonal variation. As mentioned before, sampling was performed in a well-established meadow with 100% *Z. noltei* relative coverage. The maximum Blue C stored in the Ria de Aveiro seagrass meadows was calculated considering the best available information. In 2005, the maximum relative cover of *Z. noltei* (100%) was considered, while in 2014 Blue C stock was calculated for the 50–100% range of *Z. noltei* cover, rather than for a specific value. This range accounted for the potential habitat fragmentation in some of the mapped and monitored meadows and for the low relative cover in certain *Z. noltei* meadows. Overall, in 2005, the maximum Blue C stored in Ria de Aveiro *Z. noltei* meadows was 243 Mg C, while in 2014 these meadows stored and sequestered 227 (50% cover) to 453 Mg C (maximum, 100% cover). However, it must be taken into account that a 50% cover meadow that used to be a well-established area (with 100% cover) will have an higher meaning for Blue C storage than a 50% cover meadow corresponding to a new colonised area that is still increasing its relative cover. Thus, the C stored in a former 100% cover meadow is not expected to be completely lost when a shift to 50% cover occurs, i.e., part of the stored C will still remain in the sediment.

The total C_org_ stock recorded, corresponding to the mean C_org_ stock on a per area basis, (200.3 ± 15.8 g C m^−2^; 10 cm depth; sediment and seagrass) was similar to the minimum value obtained for the sediment C_org_ stock recorded in three subarctic *Zostera marina* meadows, ranging from 197 g C m^−2^ to 595 g C m^−2^ (top 10 cm), located in three fjords of the Godthåbsfjordsystem (West Greenland)^6^. In the Red Sea seagrasses of Abu Dhabi, the sediment C_org_ stock of *Halodule uninervis*, *Halophila ovalis*, and *Halophila stipulacea* meadows ranged from 0.2 to 10.9 kg C_org_ m^−2^ ^53^ (1 m top layer), which corresponds to 20–1090 g C_org_ m^−2^ in the top 10-cm layer (although bearing in mind the limitations of this correspondence). The sediment C_org_ obtained for *Z. noltei* in Ria de Aveiro was lower than that estimated for other seagrass species (*Thalassia hemprichii*, *Enhalus acoroides*, *Halophila stipulacea*, *Thalassodendrum ciliatum*, and *Halodule uninervis*) in the Red Sea, Saudi Arabia, wherein an average of 3.4 ± 0.3 kg C_org_ m^−2^ in the top 1 m sediment layer was recorded, corresponding to 340 g C_org_ m^−2^ in the top 10 cm layer^7^. In Shark Bay (western Australia), an average of 12.8 kg C_org_ m^−2^ was obtained for the top 1 m sediment layer (1.28 kg C_org_ m^−2^ in the top 10 cm sediment layer, through direct conversion), and 50% of the sediment C_org_ stocks assessed ranged from 9.2–16.1 kg C_org_ m^−2^ (0.92–1.61 kg C_org_ m^−2^ in the top 10-cm sediment layer, through direct conversion)^15^. Thus, the results of the present study highlight the importance of seagrass meadows for storing Blue C and reducing the CO_2_ concentration in the atmosphere, which in turn contributes to climate regulation and other paramount ecosystem services including habitat provisioning, sediment stabilization, and water quality improvement. Therefore, it is crucial to preserve seagrass meadow ecosystems and to promote their recovery, as they contribute to the health of the entire ecosystem. The 2014 seagrass map obtained for Ria de Aveiro will be further improved regarding image resolution and by using multispectral imagery, to better assess the presence of seagrasses and estimate the relative coverage of the meadows. This will enhance seagrass mapping accuracy, allowing a better understanding of the ecological and physical changes occurring at this coastal lagoon system, particularly under the foreseen management interventions.

## Conclusions

Previous relevant work on the characterization and mapping of seagrass meadows was performed at Ria de Aveiro. However, this is the first study comprising extensive seagrass meadows mapping and Blue C stock assessment at this coastal lagoon, establishing a reference for seagrass meadows location, extent, and Blue C stock. Overall, Ria de Aveiro *Z. noltei* intertidal meadows seem to contradict the global decline trend, and corroborate a recent declining trend reversal observed in Europe, thereby contributing to the effective increase of the Blue C stock, and ultimately, to climate regulation and ecosystem health. This recovery over the 2003–2005 to 2013–2014 period might be related to a natural adjustment to past human interventions in the system, namely large-scale dredging activities, combined with a more accurate assessment of seagrass meadow areas due to the use of the UAV. The present study establishes a new baseline to assess the impact of the major dredging activities that are foreseen in the management plan of Ria de Aveiro, which can again endanger the recovery trend observed for *Z. noltei* intertidal meadows.

## Supplementary information

Blue Carbon stock in Zostera noltei meadows at Ria de Aveiro coastal lagoon (Portugal) over a decade

## Data Availability

The datasets generated and/or analysed during the current study are available on reasonable request.
