# Supplementary material for: Blue Carbon stock in Zostera noltei meadows at Ria de Aveiro coastal lagoon (Portugal) over a decade
Source: Sci Rep. 2019 Oct 7;9:14387. doi: 10.1038/s41598-019-50425-4 (PMC6779869; doi:10.1038/s41598-019-50425-4)

**Blue Carbon stock in *Zostera noltei* meadows at Ria de Aveiro coastal lagoon (Portugal) over a decade**

Ana I. Sousa^1*^, José Figueiredo da Silva^2^, Ana Azevedo^3^, Ana I. Lillebø^1^

^1^ Department of Biology & CESAM – Centre for Environmental and Marine Studies, University of Aveiro, Campus Universitário de Santiago, 3810-193 Aveiro, Portugal

^2^ Department of Environment and Planning, University of Aveiro, Campus Universitário de Santiago, 3810-193 Aveiro, Portugal

^3^ Department of Physics & CESAM – Centre for Environmental and Marine Studies, University of Aveiro, 3810-193 Aveiro, Portugal

* Correspondence and requests for materials should be addressed to A.I.S. (e-mail: [anaisousa@ua.pt](mailto:anaisousa@ua.pt))

**Supporting information**

Supplementary Figure S1 – *Zostera noltei* C content (% DW) for shoots and roots & rhizomes (left graph) and sediment total C, C_org_ and C_inorg_ content (right graph) throughout 2012/2013, at site M (Mira channel).


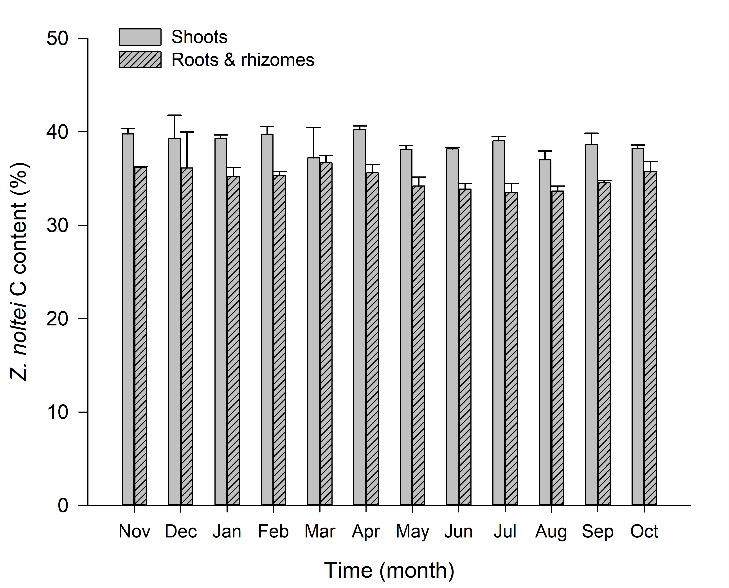

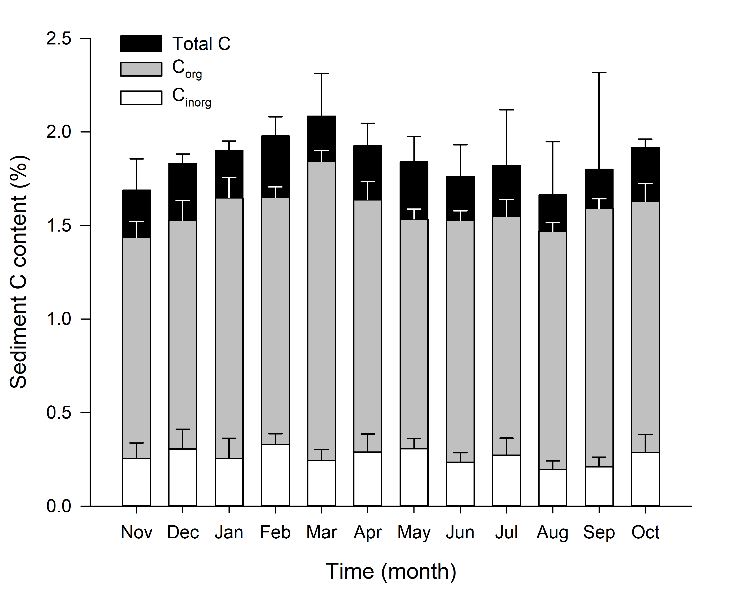


Supplementary Figure S2 - Timeline of the most relevant events and changes occurring at Ria de Aveiro coastal lagoon from the 1800’s until nowadays.
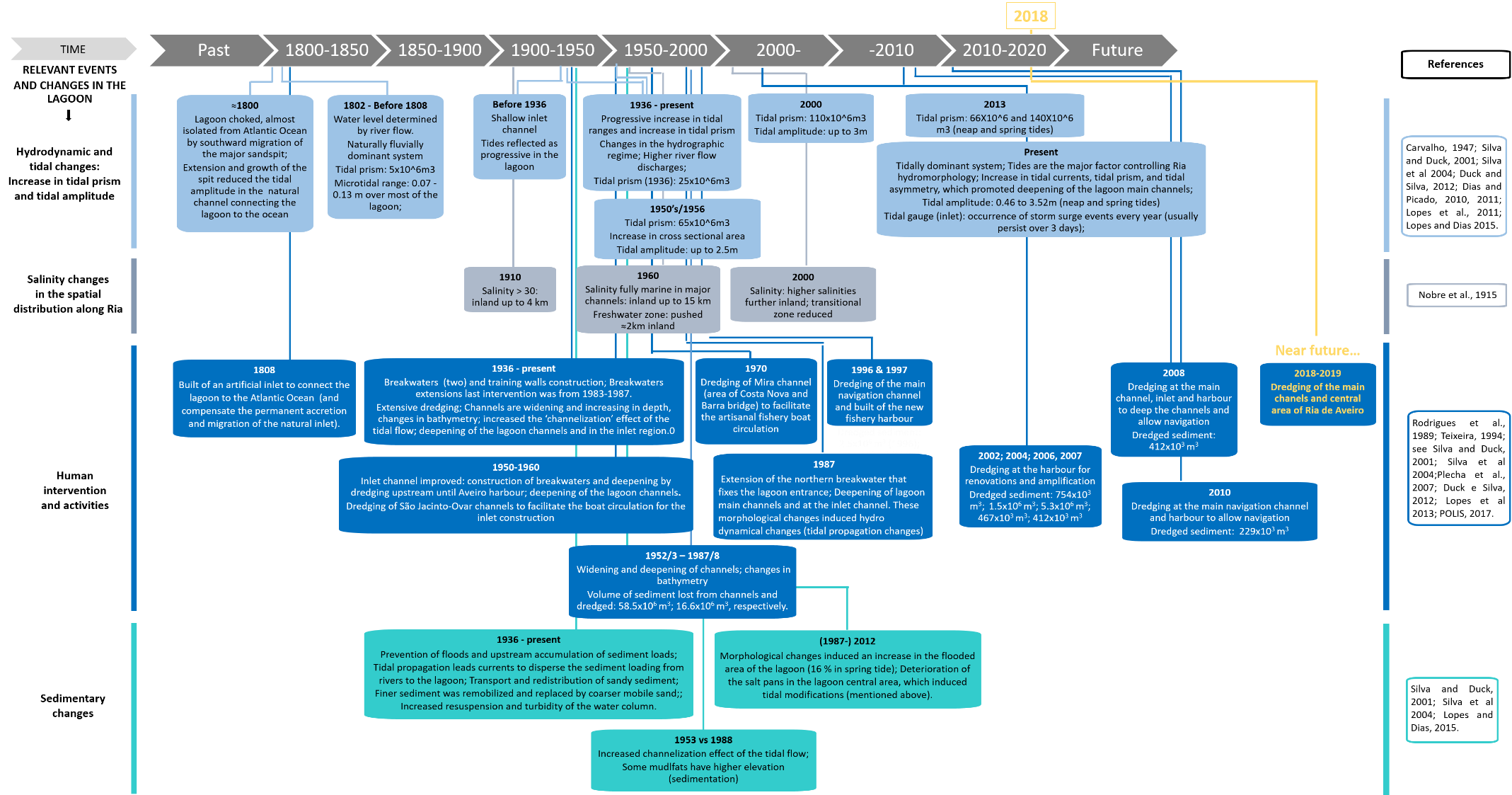

Supplement: Supplementary file 1 — Blue Carbon stock in Zostera noltei meadows at Ria de Aveiro coastal lagoon (Portugal) over a decade [file 41598_2019_50425_MOESM1_ESM.docx]
